# Seasonal Variation in Female Mate Choice and Operational Sex Ratio in Wild Populations of an Annual Fish, *Austrolebias reicherti*

**DOI:** 10.1371/journal.pone.0101649

**Published:** 2014-07-16

**Authors:** Carlos Passos, Bettina Tassino, Federico Reyes, Gil G. Rosenthal

**Affiliations:** 1 Sección Etología, Facultad de Ciencias, Universidad de la República, Montevideo, Uruguay; 2 Department of Biology, Texas A&M University, College Station, Texas, United States of America; 3 Centro de Investigaciones Científicas de las Huastecas “Aguazarca,” Calnali, Hidalgo, México; Columbia University, United States of America

## Abstract

The intensity of mating competition and the potential benefits for female of mating with certain males can be influenced by several extrinsic factors, such that behavioral decisions can be highly context-dependent. Short-lived species with a single reproductive season are a unique model to study context-sensitive mating decisions. Through exhaustive sampling in the field and simultaneous choice tests in the laboratory, we evaluated operational sex ratio (OSR) and female mate choice at the beginning and end of the reproductive season in the annual killifish *Austrolebias reicherti*. We found seasonal change in both OSR and female mate choice. At the start of the reproductive season the OSR did not deviate from parity, and females preferred larger males. Later in the reproductive season, while the proportion of males in the ponds decreased, females became unselective with respect to male size. The particular biological cycle of annual killifish, where both life expectancy and mating opportunities decline sharply over a short timescale, could account for the seasonal change in female choice. Reduction in choosiness could arise from diminished reproductive prospects due to a decline in male availability. Moreover, as the end of the season approaches, any benefits of choosiness are presumably reduced: a female’s fitness will be higher if she mates with any male than if she forgoes reproduction and dies. Future work will disentangle the mechanisms underlying seasonal changes in mating preferences, notably direct responses to demographic factors, environmental cues, or intrinsic changes during development.

## Introduction

Sexual selection results in differential reproductive success among individuals of the same sex [1]. Usually, males have a higher potential reproductive rate than females, and are thus most often the competing sex, while females are the more choosy sex (reviewed in [2]). Recent studies have shown that both the intensity of mating competition and the potential benefits for females of mating with certain males can be influenced by several extrinsic factors, such that behavioral decisions can be highly context-dependent (e.g. [3]–[6]).

Animals that expend their entire reproductive effort over the course of a single season provide a distinctive opportunity to study context-sensitive mating decisions. In this study, we examined mate choice in the annual killifish *Austrolebias reicherti* (Teleostei: Rivulidae). These fish live in temporary ponds that dry out completely during the dry summer months; lifespan is restricted by the duration of the pond, usually between 4 and 6 months [7]. *Austrolebias,* along with some other cyprinodontiform fishes, deposit desiccation-resistant eggs in the substrate of the temporary ponds they inhabit [8]. Developing embryos can undergo reversible diapauses until environmental conditions for egg eclosion become suitable [9]–[10], allowing them to survive long periods of desiccation. Individuals then hatch, mature, reproduce, and die within the span of a very short season. They therefore complete their life cycle in <1 year, with no overlapping generations.

*Austrolebias* are highly sexually dimorphic: males are larger, exhibit bright coloration, perform elaborate courtship displays, and compete for mating; while females are smaller, relatively cryptic, and do not court [11]–[12]. Female *Austrolebias* prefer to mate with larger males [13]. There are two, perhaps mutually reinforcing reasons why we might expect this preference to decline over the course of a breeding season. First, as the end of the season approaches, any benefits of choosiness are presumably reduced: a female’s fitness will be higher if she mates with any male than if she forgoes reproduction and dies. Second, females should become less choosy as the operational sex ratio (OSR) becomes increasingly female-skewed. Adult males are likely to suffer higher mortality due to exhaustion resulting from social interactions [14]–[15], a higher susceptibility to parasites and diseases [14]–[16], and increased predation risk [17]–[18]. The OSR is therefore likely to become increasingly female-biased over the course of a season. Female choosiness should be lower under a female-biased OSR than a male-biased OSR because of the increased costs of mate rejection [19]–[20]. Decreased intersexual encounter rates also mean that females may be less able to assess male phenotypic variation [21]. Indeed, several experimental studies have found that manipulating the OSR induces changes in mating preferences in the predicted direction (e.g. [22]–[26]), and field studies have shown that the strength of female mate choice covaries with population OSR (e.g. [27]).

We predicted both a decrease in the availability of male *A. reicherti* over the course of the reproductive season, and a decrease in female preference for large body size. In this study, we measured the sex ratio in multiple wild populations, and assayed female mating decisions at the beginning and at the end of the reproductive season.

## Materials and Methods

### Study Species

*Austrolebias reicherti* is endemic to the seasonal wetlands of eastern Uruguay [7]. Like other *Austrolebias* species, males of *A. reicherti* exhibit bright coloration on the opercular region and unpaired fins and a pattern of dark vertical bands upon a light background on its body flanks, whereas females are brownish [7]. Males and females engage in courtship interactions before fully or partially burying themselves in the substrate to deposit and fertilize eggs [28]. Both sexes reproduce repeatedly during the breeding season (C Passos, personal observation).

### Sex ratio

**Figure 1:**
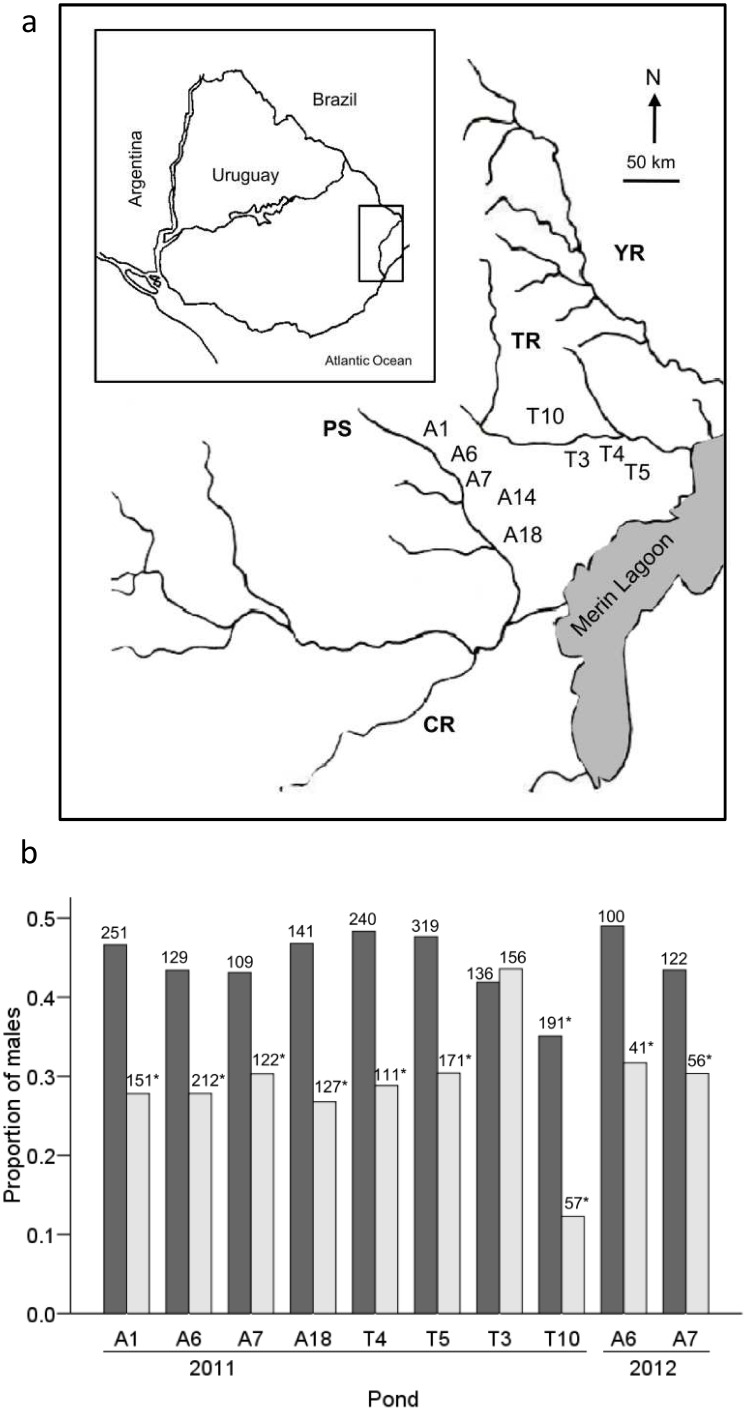
Sex ratio of *Austrolebias reicherti*. (a) Locations of temporary ponds sampled in eastern Uruguay. CR = Cebollatí River; YR = Yaguarón River; TR = Tacuarí River; PS = Parao Stream. Numbers refer to the pond numbers mentioned in the text. (b) Sex ratio (proportion male) from temporary ponds early (dark gray) and late (light gray) in the reproductive season. Numbers above the bars denote sample sizes and asterisks denote p<0.05 according to chi-squared tests. Pond codes refer to locations in map above.

Adult sex ratio was estimated from eight temporary ponds at the beginning (August) and end (October) of the reproductive season in 2011, and from two temporary ponds at the beginning (August) and end (November) of the reproductive season in 2012 (Figure 1). Each sampling was carried out by 3 persons with dip nets through a haphazard walk in the pond for 10–30 min, according to the density of fish and the size of the pond. Sex was determined by external diagnostic characters such as body pigmentation and fins shape [7]. Animals were then returned unharmed to point of capture.

### Mate choice

#### Collection and maintenance

Adult *A. reicherti* were collected from temporary ponds during the early (August) and later (November) part of the 2012 season (ponds A6, A7 and A14; Figure 1a). In both instances, test fish were kept in the laboratory for 2–5 days prior to behavioral trials under constant temperature (19°C) and natural photoperiod conditions, and were fed daily with live *Tubifex sp.* Males were kept in individual tanks (20 cm×9 cm×15 cm, length×width×height) to reduce stress from agonistic interactions and prevent the establishment of dominance hierarchies. Females were kept in communal tanks (40 cm×13 cm×15 cm) in groups of up to five individuals. Aquariums were visually isolated from each other.

#### Experimental aquarium

The experimental setup consisted of aquariums (60 cm×20 cm×20 cm) divided by two perforated transparent Plexiglas partitions, into a central compartment of 40 cm and two lateral compartments of 10 cm each. In the central compartment, three zones were defined by external markings: a central zone 30 cm long (neutral zone) and two side zones each 5 cm long (choice zones). In order to mimic the natural conditions inhabited by annual fishes, the bottom of the aquarium was completely covered with a thin layer of peat moss and *Myriophyllum* sp., a plant especially abundant in temporary ponds (C Passos, personal observation), was placed in the two lateral compartments and in the neutral zone. To reduce any external disturbance and provide uniform background, opaque screens covered both side walls and the rear wall of the aquarium. All experiments were performed between 8∶00 and 14∶00 local time.

#### Behavioral trials

We conducted mate choice tests at the beginning (n = 30) and end (n = 30) of the reproductive season, in which females were allowed to choose between a pair of males. Each individual was used only once. Females were individually placed in the central compartment and allowed to move freely during 20 min for acclimatization. They were then enclosed in a central opaque chamber (10×10×20 cm), and both males were placed randomly one in the right and the other one in the left compartment. Test females and stimulus males originated from the same pond. Vegetation partially prevented males from seeing each other therefore reducing agonistic interactions between them. After 10 min the opaque chamber was gently removed. We videorecorded four 15 min periods, separated by 1 h intervals, to assay courtship interactions. In order to ensure that females were motivated to mate, only females that remained more than 20% of the time in choice zones were retained for analysis (5 out of 30 trials were discarded in August, 3 out of 30 in November).

After all behavioral trials were completed, the left flank of all fish was photographed in an aquarium of 15 cm×10 cm×12 cm, where they were gently immobilized between a sponge and the aquarium’s wall. Digital images were used to measure standard length (anterior margin of head to distal margin of caudal peduncle).

### Variables and statistical analyses

The sex ratio was expressed as proportions of males. For each pond, deviations from the expected sex ratio of 0.5 were analyzed using chi-square tests. Sex ratio at the beginning and end of the reproductive season was compared using Wilcoxon signed-rank tests. Female preference was measured as the time spent interacting with each male in a simultaneous choice assay [13], [29]–[31]. Interaction behavior includes female orientation to the stimulus male, joint swimming, following acceptance and diving acceptance, as detailed in García et al. [28]. As a measure of female general responsiveness to the stimuli, we calculated female motivation, defined as the total interaction time with both males in the choice zones. Because a female’s size could affect her general propensity to interact with males [32], we evaluated the potential relationship between female size and female motivation by Pearson correlation. Female motivation at different times of the reproductive season was compared using a two-sample t-test. A particular male was considered preferred if the female interacted with him more than 50% of the total interaction time [13], [33]. To evaluate the effect of male phenotype on females’ mating decisions, we compared the body size of preferred and non-preferred males at the beginning and end of the reproductive season using a paired t-test.

We measured the strength of preference as the net difference in the time females spent with the preferred versus the non-preferred male [34]. We used linear regression to examine whether male body size predicted the strength of female preference by regressing the difference in time spent with each male against the corresponding differences in body size between the two males. We also tested for seasonal differences in both the strength of female preference and male size asymmetry (standard length of large male – standard length of small male) during the early and later part of the reproductive season using two-sample t-tests. Data were checked for normality using Kolmogorov–Smirnov tests. Reported values are mean ± standard error. Statistical analyses were performed using SPSS, version 15.0 for Windows (SPSS Inc., Chicago, IL, USA).

### Ethics statement

Collection and experimental procedures were approved by the ethical committee of the Universidad de la República, Uruguay (Comisión de ética en el uso de animales, Facultad de Ciencias, UdelaR, ANII FCE 241000-001186-12). After completing the mate choice experiments, all fish were retained as breeding stock.

## Results

### Sex ratio

Altogether 2942 individuals (1141 males and 1801 females) were collected throughout the season. The proportion of males in the ponds decreased throughout the reproductive season (early = 0.46±0.01, late = 0.29±0.02; Wilcoxon signed rank test: Z = −2.705, N = 10, *P* = 0.007). At the beginning of the reproductive season, the sex ratio only deviated from parity in one pond. However, females dominated in 9 out of 10 ponds at the end of the reproductive season (Figure 1b).

### Mate choice

Overall, females spent most of their time in the choice zones (71.5±2.6%) oriented to and interacting with one of the males. The interaction time that females spent in the choice zones (both sides combined) did not differ significantly at the beginning and end of the reproductive season (two-sample t-test: *t_50_* = −0.146, *N1* = 25, *N2* = 27, *P* = 0.884), and there was no relationship between time spent interacting with males and female size (Pearson correlation: *r_52_* = 0.144, *P* = 0.310). Moreover, neither the strength of preference nor the asymmetry in size between males were significantly different between the two periods (strength of preference: early = 996±128 s, late = 1102±171 s; two-sample t-test: *t_50_* = −0.490, *N1* = 25, *N2* = 27, *P* = 0.626; asymmetry in size between males: early = 3.91±0.55 mm, late = 2.98±0.49; two-sample t-test: *t_50_*:  = 1.299, *N1* = 25, *N2* = 27, *P* = 0.200).

**Figure 2:**
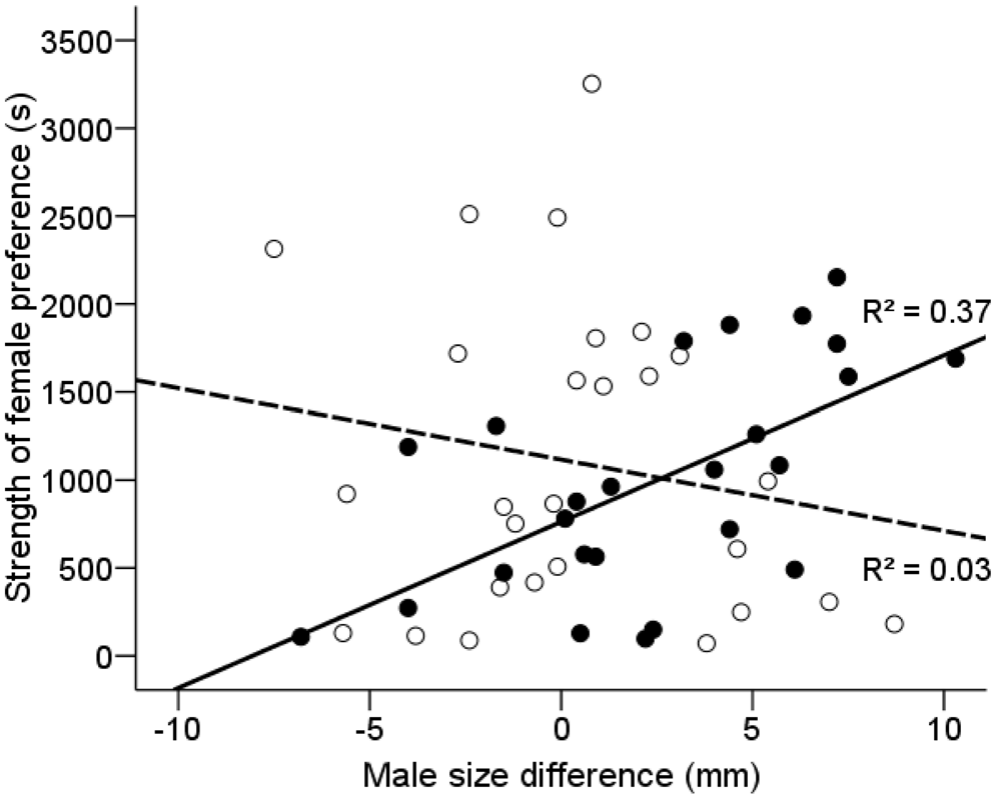
Female mate choice and male body size. Relationship between male size difference (standard length of preferred male − standard length of non-preferred male) and strength of female preference (difference in time spent with preferred and non-preferred male) in the annual killifish *A. reicherti* at the beginning (black circles, black line) and end (white circles, dashed line) of the reproductive season. Negative scores indicate more time with smaller male, positive scores indicate more time with larger male.

At the beginning of the reproductive season, females preferred larger males (preferred males = 36.53±0.86 mm, non-preferred males = 34.06±0.77 mm; paired t-test: *t_24_* = 2.987, *P* = 0.006). The strength of female preference was a positive function of the difference in male size (*r^2^* = 0.371, *F_1,23_* = 13.573, *P* = 0.001; Figure 2). At the end of the reproductive season, the body size of preferred and non-preferred males did not differ significantly (preferred males = 39.06±0.82 mm, non-preferred males = 38.71±0.79 mm; paired t-test: *t_26_* = 0.467, *P* = 0.645). Moreover, the strength of preference was not associated with the difference in male size (*r^2^* = 0.031, *F_1,25_* = 0.806, *P* = 0.373; Figure 2; Table S1).

## Discussion

We found seasonal change in both operational sex ratio and female mate choice in the annual killifish *Austrolebias reicherti*. At the beginning of the reproductive season (August) the OSR did not deviate from parity, and females preferred larger males. Later in the reproductive season (October–November), the OSR became female-biased and females became unselective with respect to male size.

These results are consistent with previous findings showing that female choosiness decreases under female-biased OSR [22]–[27]. Declines in the number of males, leading to female-biased sex ratios late in the breeding season, have been reported in other fish (e.g. [5]), including annual killifish in the genus *Nothobranchius* [35]. We have not observed any sex differences in mortality rate or biases in the adult sex ratio in the laboratory, suggesting that increased extrinsic mortality in males could lead to the observed biases in the OSR at the end of the season in natural populations. *Austrolebias* species exhibit both intense male–male competition, often involving serious injuries to subordinate males, and elaborate male courtship displays during reproduction [13], [28]. A plausible explanation is that high male mortality stems from exhaustion resulting from courtship displays or male-male competition [14]–[15]. Further, males exhibit bright coloration and showy sexual displays that may incur increased predation risk [17], [36].

At the start of the reproductive season, larger males were strongly preferred, and the strength of female preference was positively related with the size difference between stimulus males. Preference exhibited by female *A. reicherti* towards larger males is consistent with female choice based on male body size, which is widespread in cyprinodontiform fishes [37]–[39]. Passos et al. [13] previously reported that females preferred larger males in *A. charrua*.

However, female preference for larger males vanished at the end of the reproductive season. Females were equally likely to choose small or large males later in the season and female preference was not affected by the size difference between stimulus males. It is important to note that male size distributions, and the mean differences between tested males, did not change over the course of the breeding season. The observed change in preference for body size is therefore the result of changes in female choosiness rather than the distribution of available males.

Reduction in choosiness could arise from diminished reproductive prospects due to a decline in male availability [40]–[41]. As the sex ratio becomes female biased late in the season, the opportunity for female choice may be reduced by a decline in rate of encounters with males [42], an increase in female-female competition, and/or because males may become more selective in their mating decisions [5]. Further, approaching mortality may cause time constraints on female mate searching [43]–[44]. As this critical time approaches, a female may be faced with a trade-off in which she will have to decrease choosiness or risk losing the opportunity to fertilize her eggs. In this regard, the particular biological cycle of annual killifish, where both life expectancy and mating opportunities decline over time, could account for the seasonal change in female choice.

Although we have shown that female preference for body size is reduced over the course of the season, it is not necessarily the case that female *A. reicherti* become entirely non-selective. In fact, neither the difference in the time females spent with each male nor the total interaction time with both males were significantly different at the beginning and end of the reproductive season. Then, the observed seasonal variation in female choice may be due to shifts in the weights that females assign to different male traits [3]. The fitness consequences for females of mating with certain males may vary throughout the season, and females might then be expected to change how they respond to multiple male cues as environmental conditions or individual phenotypes change (e.g. [45]–[46]). Larger males have a competitive advantage with respect to male-male competition [13] and females could obtain direct benefits from mating with them early season to gain access to preferred areas for oviposition. However, the advantages of male size and the benefits to females of choosing large males may be reduced late in the season because lower male density may decrease the intensity of male-male competition. These reduced direct benefits are a potential driver of the lack of preference for male size at the end of the reproductive season. Therefore, female choice in *A. reicherti* could be based on other cues if traits other than male size become relatively more important for female reproductive success later in the reproductive season. Females do not attend to variation in male pigmentation (unpublished data), but Passos et al. [47] showed that female *A. reicherti* respond to the chemical cues of potential mates. The habitat of annual fish such as *A. reicherti* consists mostly of shallow ponds with muddy substrate, turbid water and high vegetation density, which could hinder the use of vision particularly near the end of a season. The extremely variable habitat provided by temporary ponds exposes *A. reicherti* to drastic environmental changes, and the use of different sensory modalities could vary over the life cycle (see [48]–[49]). Future studies will address whether sensitivity to chemical cues changes over the course of a season.

In summary, we have shown seasonal changes in both operational sex ratios, and mating preferences for male body size in the annual killifish *A. reicherti*. Female preference for larger males disappeared with the decline of male availability over the season and as the window of opportunity for future reproduction became narrower. Future work will address whether females attend to different male traits over the course of a season, and disentangle the mechanisms leading to female behavioral changes, notably direct responses to demographic factors, environmental cues, or intrinsic changes during senescence.

## Supporting Information

Table S1 **Standard length of focal females and stimulus males, and females responses in simultaneous choice trials.**
(DOCX)
