# Supplementary material for: Seasonal Variation in Female Mate Choice and Operational Sex Ratio in Wild Populations of an Annual Fish, Austrolebias reicherti
Source: PLoS One. 2014 Jul 16;9(7):e101649. doi: 10.1371/journal.pone.0101649 (PMC4100733; doi:10.1371/journal.pone.0101649)
Supplement: Table S1 — Standard length of focal females and stimulus males, and females responses in simultaneous choice trials. (DOCX) [file pone.0101649.s001.docx]

|  | Standard length of female (mm) | Standard length of males (mm) | | Female interaction time (s) | |
| --- | --- | --- | --- | --- | --- |
|  |  | Preferred male | Non preferred male | Preferred male | Non preferred male |
| August | 30,4 | 38,4 | 32,3 | 1491 | 1000 |
|  | 29,9 | 35,4 | 27,9 | 2388 | 801 |
|  | 33,5 | 38,3 | 32,6 | 2105 | 1021 |
|  | 29,1 | 40,4 | 36,0 | 2240 | 359 |
|  | 30,8 | 41,0 | 36,6 | 1472 | 751 |
|  | 32,9 | 27,4 | 34,2 | 875 | 767 |
|  | 33,9 | 32,6 | 30,2 | 1713 | 1564 |
|  | 29,9 | 35,8 | 34,9 | 1449 | 884 |
|  | 29,3 | 32,9 | 32,5 | 1344 | 466 |
|  | 33,5 | 35,4 | 31,4 | 1535 | 475 |
|  | 32,7 | 43,4 | 36,2 | 2359 | 207 |
|  | 31,9 | 43,3 | 41,1 | 1537 | 1439 |
|  | 30,4 | 30,0 | 31,7 | 1347 | 39 |
|  | 33,5 | 42,5 | 32,2 | 1930 | 241 |
|  | 32,5 | 33,8 | 35,3 | 1776 | 1302 |
|  | 34,0 | 42,4 | 35,2 | 2460 | 686 |
|  | 29,6 | 33,2 | 26,9 | 2498 | 565 |
|  | 29,8 | 37,0 | 41,0 | 1407 | 218 |
|  | 32,3 | 38,4 | 38,3 | 1525 | 744 |
|  | 33,6 | 32,6 | 36,6 | 1744 | 1472 |
|  | 32,7 | 35,4 | 34,9 | 1670 | 1542 |
|  | 35,6 | 32,5 | 27,4 | 2145 | 884 |
|  | 30,6 | 34,2 | 32,9 | 1603 | 640 |
|  | 30,7 | 35,8 | 32,6 | 2074 | 285 |
|  | 32,5 | 41,2 | 40,6 | 1762 | 1185 |
| November | 34,1 | 38,7 | 41,1 | 1364 | 1276 |
|  | 30,8 | 31,8 | 32,5 | 720 | 302 |
|  | 32,3 | 39,8 | 45,4 | 1738 | 817 |
|  | 32,2 | 38,0 | 37,2 | 3365 | 112 |
|  | 32,3 | 40,4 | 46,1 | 1497 | 1367 |
|  | 36,0 | 41,3 | 39,0 | 1664 | 75 |
|  | 29,3 | 38,2 | 34,4 | 336 | 265 |
|  | 32,0 | 32,8 | 34,4 | 1300 | 910 |
|  | 33,0 | 43,6 | 43,2 | 1814 | 250 |
|  | 31,9 | 44,6 | 44,8 | 1985 | 1119 |
|  | 32,7 | 35,4 | 34,3 | 2473 | 941 |
|  | 32,1 | 44,6 | 39,2 | 1894 | 900 |
|  | 37,1 | 41,3 | 41,4 | 1076 | 568 |
|  | 31,9 | 43,6 | 40,5 | 2404 | 699 |
|  | 32,0 | 42,2 | 35,2 | 1800 | 1493 |
|  | 31,4 | 35,7 | 38,4 | 1750 | 32 |
|  | 31,8 | 48,1 | 43,5 | 1726 | 1118 |
|  | 31,1 | 38,5 | 40,0 | 1189 | 341 |
|  | 32,7 | 41,6 | 39,5 | 2496 | 654 |
|  | 31,1 | 35,0 | 36,2 | 1688 | 936 |
|  | 31,9 | 35,8 | 39,6 | 1680 | 1566 |
|  | 30,2 | 42,2 | 33,5 | 1829 | 1648 |
|  | 27,8 | 39,1 | 34,4 | 1415 | 1166 |
|  | 30,3 | 32,0 | 39,5 | 2571 | 257 |
|  | 32,0 | 35,3 | 34,4 | 2643 | 838 |
|  | 32,0 | 33,5 | 33,6 | 2816 | 325 |
|  | 34,4 | 41,6 | 44,0 | 2581 | 70 |

Table S1. Standard length of focal females and stimulus males, and females responses in simultaneous choice trials
